# Reorganization and Stability for Motor and Language Areas Using Cortical Stimulation: Case Example and Review of the Literature

**DOI:** 10.3390/brainsci3041597

**Published:** 2013-11-26

**Authors:** Sandra Serafini, Jordan M. Komisarow, William Gallentine, Mohamad A. Mikati, Melanie J. Bonner, Peter G. Kranz, Michael M. Haglund, Gerald Grant

**Affiliations:** 1 Division of Neurosurgery, Department of Surgery, Duke University Medical Center, Box 3807, Durham, NC 27710, USA; E-Mails: jordan.komisarow@dm.duke.edu (J.M.K.); michael.haglund@dm.duke.edu (M.M.H.); 2 Division of Pediatric Neurology, Department of Neurology, Duke University Medical Center, Box 3936, Durham, NC 27710, USA; E-Mails: william.gallentine@dm.duke.edu (W.G.); mohamad.mikati@dm.duke.edu (M.A.M.); 3 Department of Psychiatry and Behavioral Sciences, Duke University Medical Center, Box 3527, Durham, NC 27705, USA; E-Mail: melanie.bonner@dm.duke.edu; 4 Division of Neuroradiology, Department of Radiology, Duke University Medical Center, Box 3808, Durham, NC 27710, USA; E-Mail: peter.kranz@duke.edu; 5 Department of Neurosurgery, Stanford University, Lucile Packard Children’s Hospital, R211 MC 5327, 300 Pasteur Drive, Stanford, CA 94305, USA; E-Mail: ggrant2@stanford.edu

**Keywords:** language, motor, cortical stimulation, reorganization, pediatric

## Abstract

The cerebral organization of language in epilepsy patients has been studied with invasive procedures such as Wada testing and electrical cortical stimulation mapping and more recently with noninvasive neuroimaging techniques, such as functional MRI. In the setting of a chronic seizure disorder, clinical variables have been shown to contribute to cerebral language reorganization underscoring the need for language lateralization and localization procedures. We present a 14-year-old pediatric patient with a refractory epilepsy disorder who underwent two neurosurgical resections of a left frontal epileptic focus separated by a year. He was mapped extraoperatively through a subdural grid using cortical stimulation to preserve motor and language functions. The clinical history and extensive workup prior to surgery is discussed as well as the opportunity to compare the cortical maps for language, motor, and sensory function before each resection. Reorganization in cortical tongue sensory areas was seen concomitant with a new zone of ictal and interictal activity in the previous tongue sensory area. Detailed neuropsychological data is presented before and after any surgical intervention to hypothesize about the extent of reorganization between epochs. We conclude that intrahemispheric cortical plasticity does occur following frontal lobe resective surgery in a teenager with medically refractory seizures.

## 1. Introduction

Medically refractory epilepsy in children may lead to resective surgery if the epileptogenic zone can be localized. Seizure control is the goal following surgery with or without the need for continued anticonvulsant therapy. Prior to surgery, a variety of tests and mapping techniques are completed depending on the age and cooperation level of the patient, including neuropsychological assessment, Wada testing, Single-Photon Emission Computed Tomography (SPECT), Positron Emission Tomography (PET), Diffusion Tensor Imaging (DTI), functional MRI (fMRI), and a Magnetic Encephalogram (MEG). A subdural grid array is then surgically placed on the brain to better delineate the margins of the epileptogenic zone and to perform extraoperative electrical cortical stimulation (ECS). Together, these localization techniques provide information to pinpoint the epileptic focus that should be removed as well as identify the areas for motor, sensory, and language function so they can be avoided to minimize post-operative functional deficits.

While plasticity of motor and language functions to the contralateral hemisphere have been extensively studied in pediatric patients with early acquired lesions or who have undergone functional hemispherectomies [1], the intrahemispheric organization of motor and language functions in children has received relatively less attention. Case studies in pediatric [2] and adult [3] patients with cortical dysplasia show that resections in the primary hand motor cortical areas may result in an immediate complete motor deficit. However, the deficit may be transient since a substantial recovery can still occur in children and young adults after several months of rehabilitation. Similarly, resections of language areas in pediatric patients that result in either expressive or receptive language deficits in the early postoperative period are also able to reorganize intrahemispherically [4]. This intrahemispheric cortical plasticity and postlesional functional reorganization is thought to depend on the preservation of white matter tracts, and so far it appears that damage to white matter precludes structural plasticity [5].

### White Matter Maturation and Cognitive Development

The prefrontal cortex is involved in several cognitive functions, including executive function, attention, memory, reasoning, and language comprehension. There is an extensive literature that explores the concurrent changes in neuroanatomical structure and cognitive maturation throughout childhood [6,7,8,9,10]. Changes in the prefrontal cortex of developing children include synaptic density, increased dendrites, and increases in the diameter and myelination of axons [11]. Several recent imaging studies have shown that maturation of white matter tracts, particularly during the latter part of childhood, has correlated to more discrete functional processes and abilities [12] such as the development of visuospatial working memory and reading ability [13].

In this paper, we report the case of a pediatric patient who underwent two surgical resections of a left frontal seizure focus at the ages of 12 and 13 and four serial neuropsychological assessments at the ages of 6, 10, 13 and 14. Based on past studies in cortical stimulation, the role of frontal subcortical anatomy and frontal lobe functional development, we hypothesized that we would see a mixture of stable as well as reorganized functional areas in and around the temporal and frontal lobes. Reorganization is discussed in a context of possible age-related changes *versus* changes induced by pathology or surgically-induced functional changes in frontal areas.

## 2. Results and Discussion—Case Report

### 2.1. History and Neurological Examination

**Figure 1:**
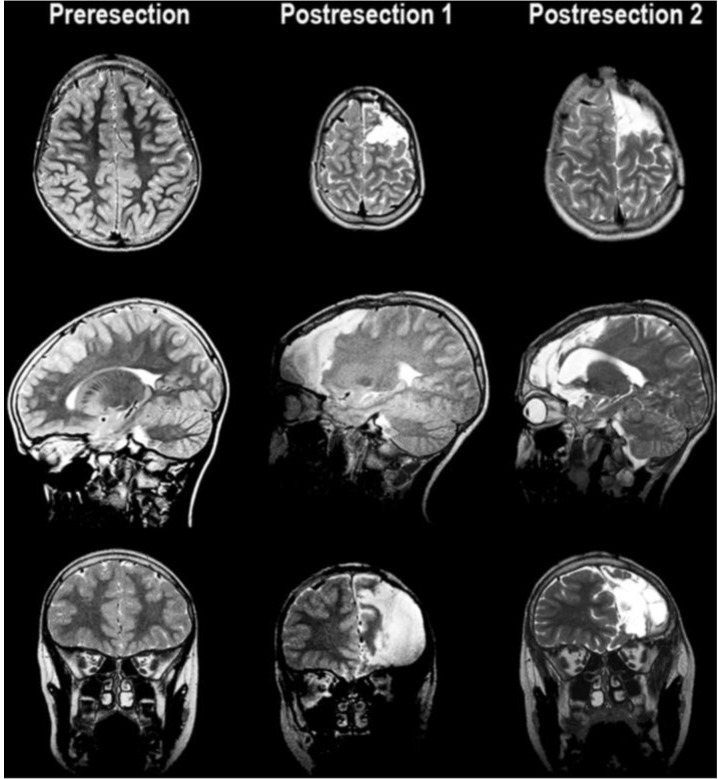
MRI images showing pre-resection (*left column*), and resection cavity following the first procedure (*middle column*) with resection to the pre-motor gyral bank and second procedure (*right column*) with resection back to the primary motor area.

This 14-year-old left-handed male originally presented at two and a half years of life with new onset seizures. Birth was via emergent cesarean section due to fetal bradycardia. APGAR (Appearance, Pulse, Grimace, Activity, Respiration scores of 0–2 each that are summed to assess the health of a newborn with a resulting score range of 0–10, with 7 and above considered normal) scores were six at one minute, and nine at five minutes of life. The patient was reportedly in good health and undergoing normal development until 18 months of age when his first seizure was observed and was characterized by elevation of his right arm. Over the course of the next few months the patient had several more similar episodes, which escalated to include hyperventilation and fits of laughter. Two ambulatory EEGs were performed. The first EEG captured a seizure described as left hemisphere dysrhythmia without spreading. The second EEG did not capture seizure activity but demonstrated interictal epileptogenic regions in the frontotemporal region (F3). Computerized Tomography (CT) scan and Magnetic Resonance Imaging (MRI) of the head were normal (see Figure 1, left column).

Because the patient did not tolerate initial management with gabapentin due to side effects, he was transitioned to a combination of lamotrigine and topiramate. At the age of 9, the patient had a vagal nerve stimulator installed due to continued medically refractory seizures, which unfortunately did not appear to have any significant effect on his seizure control. Until the age of 11, the patient was unsuccessfully medically managed at an outside institution. At the time of presentation to our institution at age 11, his seizures were reported to consist of upward and rightward eye deviation, leg kicking, gurgling, shallow breathing, and grinding of his teeth. The right arm became tonically flexed during these episodes. The patient was unresponsive during these events. These seizures most frequently occurred between midnight and six o’clock in the morning in clusters lasting approximately 15 min.

### 2.2. Neuropsychological Assessments

**Figure 2:**
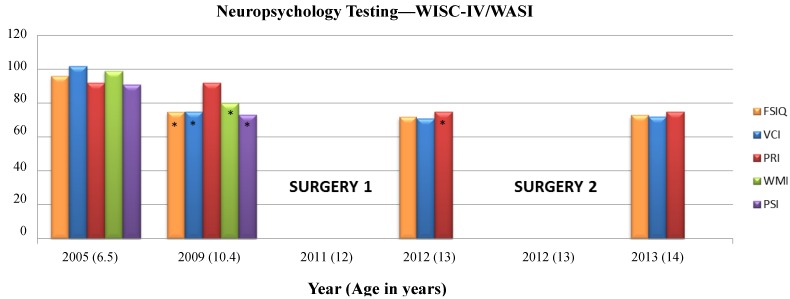
Neuropsychological testing timeline for cognitive abilities. WISC IV = Wechsler Intelligence Scale for Children-Fourth Edition; WASI = Wechsler Abbreviated Scale of Intelligence; FSIQ = Full Scale Intelligence Quotient; VCI = Verbal Comprehension Index; PRI = Perceptual Reasoning Index; WMI = Working Memory Index, PSI = Processing Speed Index. Qualitative classification ranges for standard scores [Very Superior (>129), Superior (120–129), High Average (110–119), Average (90–109), Low Average (80–89), Borderline (70–79), and Impaired (<70)]. Scores have a mean of 100 and Standard Deviation (SD) of 15. See Supplementary Information for core subtest descriptions. * Decrease by >1 standard deviation from previous assessment within index. Surgery 1 and 2 refer to the timing of the first and second surgical resections.

**Figure 3:**
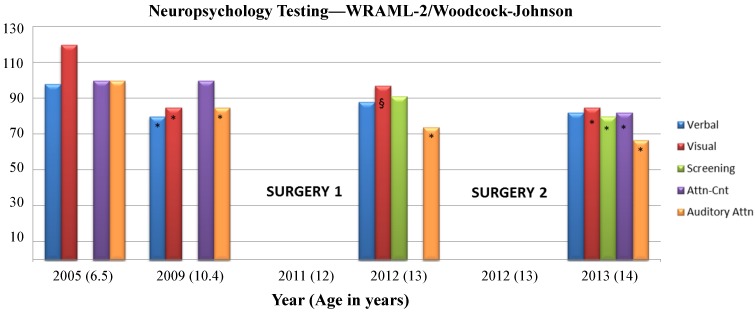
Neuropsychological testing timeline for memory and cognitive abilities. Wide Range Assessment of Memory and Learning (WRAML-2) and Woodcock-Johnson tests of Cognitive Abilities-III, qualitative classification ranges for standard scores: Very Superior (>129), Superior (120–129), High Average (110–119), Average (90–109), Low Average (80–89), Borderline/Mildly Deficient (70–79), and Impaired/Extremely Low (<70). Scores have a mean of 100 and Standard Deviation (SD) of 15. See Supplementary Information for core subtest descriptions. * Decrease by >1 standard deviation from previous assessment within index. § Increase by >1 standard deviation from previous assessment within index. Surgeries 1 and 2 refer to the timing of the first and second surgical resections.

The Wechsler Intelligence Scale for Children-Fourth Edition (WISC-IV) was administered at ages 6 years, 6 months (2005) and at 10 years, 5 months (2009) to calculate four index scores and a Full Scale IQ (FSIQ) using 10 subtests. The Verbal Comprehension Index (VCI) used the Similarities, Vocabulary, and Comprehension subtests. The Perceptual Reasoning Index (PRI) used the Block Design, Picture Concepts, and Matrix Reasoning subtests. The Working Memory Index (WMI) used the Digit Span and Letter-Number Sequencing subtests, and the Processing Speed Index (PSI) used the Coding and Symbol Search subtests. The Wechsler Abbreviated Scale of Intelligence (WASI) is organized into Verbal Scale (Similarities and Vocabulary subtests) and Performance Scale (Block Design and Matrix Reasoning subtests) composites that also give a Full Scale Intelligence Quotient—the WASI was administered at ages 13 years, 1 month (2012) and 14 years, 1 month (2013) in place of the full WISC-IV to yield VCI, PRI, and FSIQ scores. Scores have a mean of 100 and Standard Deviation (SD) of 15, they are represented in Figure 2. The Wide Range Assessment of Memory and Learning (WRAML-2) was used to assess memory functioning. Four core subtests were administered to provide information regarding verbal and visual memory abilities, and a composite score, the Screening Memory Index, was also calculated to provide information about general memory functioning (Figure 3). See Supplementary Information for descriptions of each subtest. At the time of each evaluation, a positive medication response to Ritalin was seen in treating and improving secondary ADHD symptoms.

Noted trends were that prior to the first surgery, almost all cognitive domain scores decreased by more than one standard deviation from 2005 to 2009 (from Average to Low Average/Borderline) with the exception of the PRI, representing stability in analyzing and synthesizing abstract visual stimuli, abstract categorical reasoning ability, and general nonverbal intelligence. Declines in VCI, WMI, PSI, and FSIQ were concurrent with increases in seizure frequency and decreases in responsiveness to anti-epileptic medications. Lower VCI scores represented declines in verbal reasoning and concept formation, auditory comprehension, and verbal expression. Lower WMI scores represented declines in auditory short-term memory, sequencing skills, attention/concentration, visual-spatial imaging, and mental manipulation, and lower PSI scores represented declines in speed of visual scanning, cognitive flexibility, visual-motor coordination, short-term visual memory, visual discrimination, and concentration. Following the first surgical partial frontal lobe resection, the PRI index decreased by more than one standard deviation (Low Average to Borderline) representing a decrease in analyzing and synthesizing abstract visual stimuli, abstract categorical reasoning ability, and general nonverbal intelligence. Scores for the FSIQ, VCI, and PRI remained stable following the second surgical resection.

Memory and executive function showed similar trends in that verbal and visual memory and auditory attention saw declines between 2005 and 2009, while attention-concentration scores remained stable. The Verbal Memory Index estimates how well meaningful verbal information and relatively rote verbal information can be learned and recalled. The Visual Memory Index estimates how well meaningful (*i.e.*, pictorial) and minimally related, rote (*i.e.*, design) visual information can be learned and recalled; both indices have implications for both daily and academic tasks. Auditory attention measures speech-sound discrimination, including the ability to overcome the effects of auditory distortion or masking in understanding oral language. Following the first surgical resection, auditory attention continued to decline, while verbal memory remained stable and visual memory improved. Following the second surgical resection, verbal memory again remained stable while all other memory and executive function domains declined to borderline or impaired levels.

### 2.3. Grid Mapping 1

**Figure 4:**
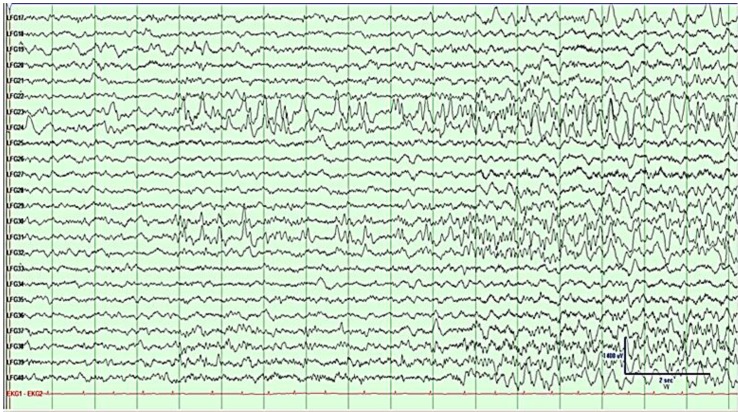
Electrocorticography (ECoG) of grid rows 3–5, prior to first resection. Activity shows left frontal seizure onset at LFG (left frontal grid) 23–24 and 30–31 characterized by semi-rhythmic sharp activity evolving into faster frequencies, followed by spread. See bottom right for μV/time reference.

**Figure 5:**
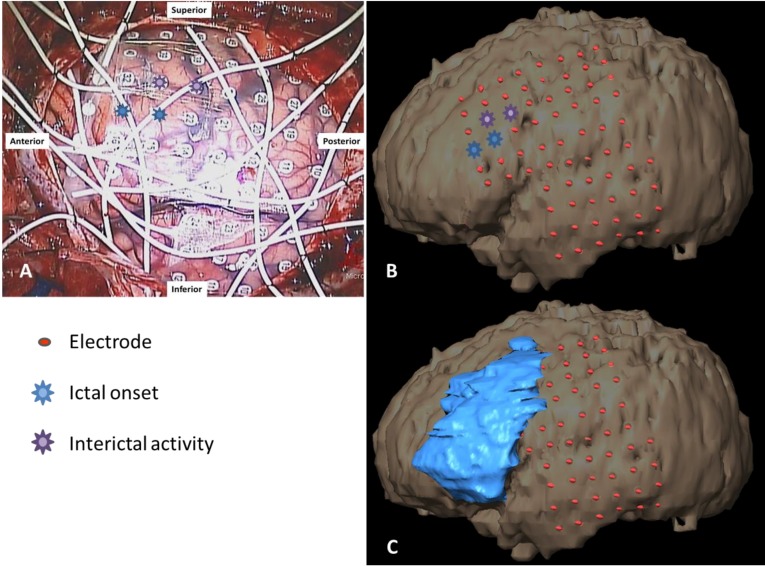
(**A**) Grid placements with anterior 1 × 6 strip shown anterior to the main frontal grid intraoperatively; (**B**) overlaid on a 3D reconstruction; and (**C**) with resection cavity. Blue and purple symbols indicate main and secondary seizure foci, respectively. Four 1 × 4 and three 1 × 6 strips are not shown.

At the age of 12, the patient underwent placement of bitemporal and bifrontal strip electrodes for seizure mapping, which demonstrated that the seizures lateralized to the left frontal lobe. Three months later, the patient underwent a craniotomy to implant a 6 × 8 (48-contact) frontal and 4 × 6 (24-contact) temporal grid array for seizure localization and extraoperative mapping, with seizure onset characterized by semi-rhythmic sharp activity evolving into faster frequencies, followed by spread (Figure 4). Each grid array used 5-mm-diameter electrodes embedded in Silastic with center-to-center interelectrode distances of 1 cm. The exposed cortical surface and grid position were documented using digital photography and schematic diagrams, functional sites were marked with a sterile 5-mm^2^ tag (Figure 5A). Brainlab (iPlan Cranial 3.0, Westchester, IL, USA) was used to co-register a pre-operative MRI and post-grid CT scan to produce a three-dimensional reconstruction of the brain with overlaid grid. Post-resection MRI scans were then co-registered to demonstrate each resection cavity. (Figure 5B,C). We used a standard stimulation procedure [14] with biphasic square-wave pulses of 0.5 ms duration at 60 Hz, with a maximum train duration of 4 s. Grid contacts were stimulated systematically extraoperatively at 4–6 mA to perform mapping of motor and language function. The stimulation threshold was determined by escalating up the amplitude until reaching the afterdischarge threshold. Once this maximum was reached, the current was backed down by 1 mA so as not to elicit seizures during stimulation which would potentially interfere with the interpretation of a positive stimulation site.

Once the primary motor cortex was identified both by direct cortical stimulation as well as phase reversal of cortically evoked responses with median nerve stimulation, the patient underwent his first of two surgical resections with a partial frontal lobectomy sparing the gyral bank of the posterior portion of superior, middle, and the operculum portion of frontal gyrus, just anterior to the primary motor strip [Figure 1 (middle column)]. The white matter tracts were preserved posterior to the resection by respecting the vertical organization of the motor tracts. This resection was carried out with the assistance of intraoperative motor mapping to confirm that the primary motor tracts were spared after the resection through consistent stimulation of the motor hand area. No histopathological abnormality was found upon microscopic examination of the resected tissue.

### 2.4. Fiber Tracking

**Figure 6:**
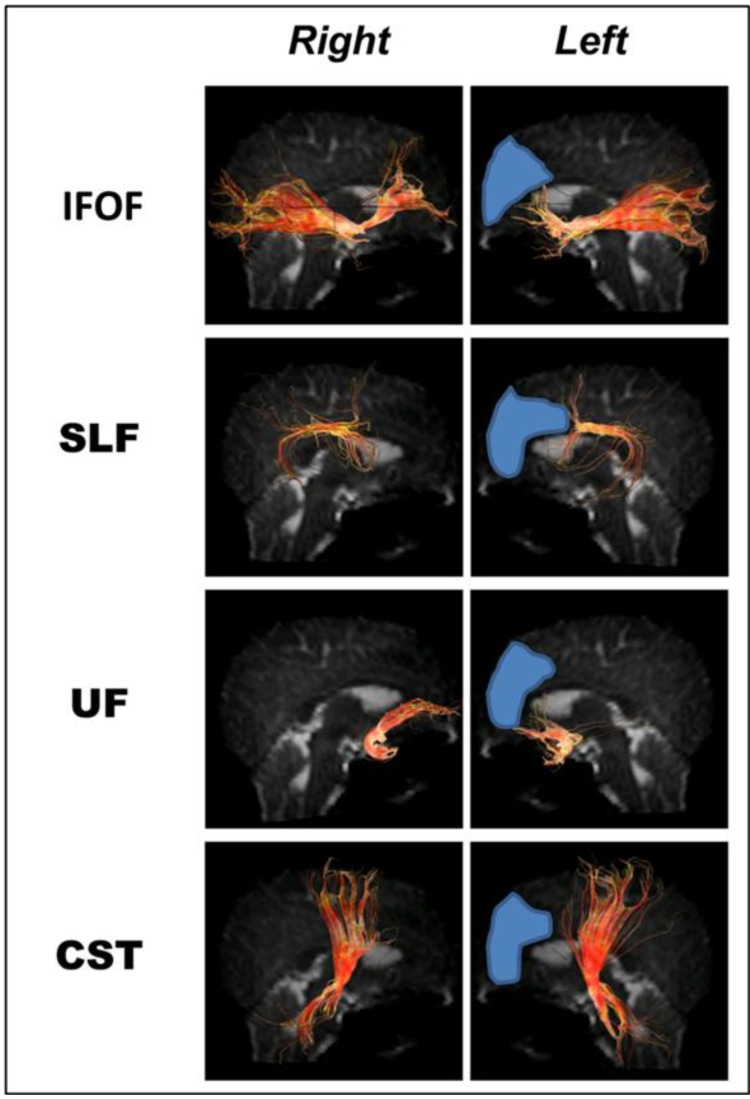
Tractography following the first surgical resection of the left frontal lobe (resection areas in *blue*) showing anterior portions of the left inferior frontooccipital fascicle (IFOF), uncuate fasciculus (UF), and corticospinal tract (CST) removed, and the projections of the long segment of the left superior longitudinal fasciculus (SLF) [*i.e.*, the arcuate fasciculus (AF)] removed. The medial portion of the SLF is preserved.

The anterior and anterosuperior portions of the inferior frontooccipital fascicle (IFOF) and its projections were resected as was a far anterior section of the corticospinal tract (CST), and an anterior portion of the arcuate fasciculus (AF) that terminated in the inferior and middle frontal gyri; the medial and posterior segments of superior longitudinal fascicle (SLF) and uncinate fascicle (UF) were preserved (Figure 6).

**Figure 7:**
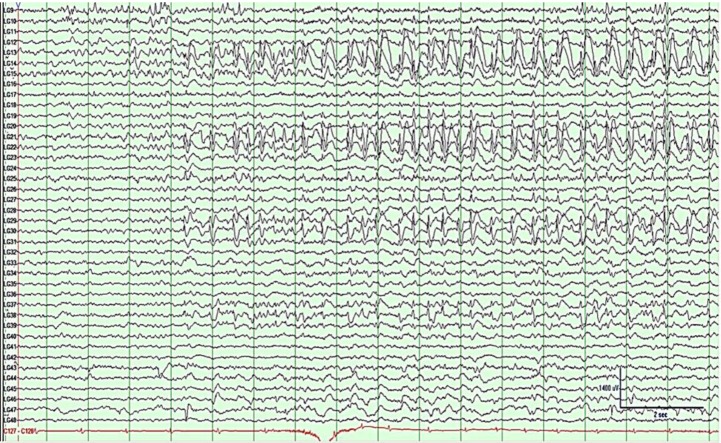
Subdural grid EEG of grid columns 1–5 prior to second resection showing left frontal seizure activity with diffuse onset at contacts 13–14, 21–22, 29–30, 37–38, characterized by rhythmic spike and slow wave activity. See bottom right for µV/time reference.

**Figure 8:**
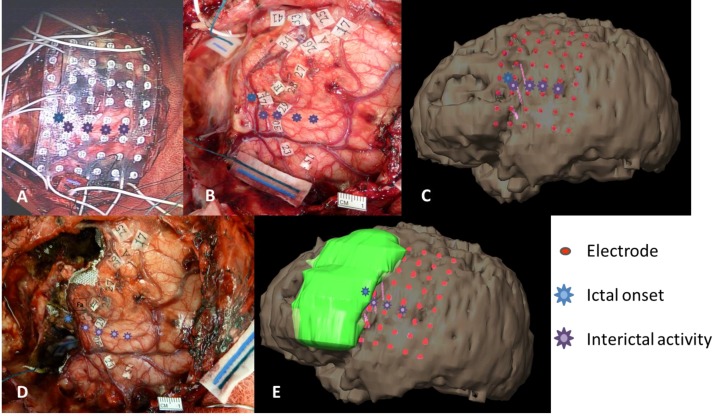
Placement of grid (**A**) and sterile functional tags (**B**) after first resection and prior to second resection as seen intraoperatively, and on 3D reconstruction (**C**). *Blue symbol* indicates seizure focus at one of three depth electrodes. Purple symbols show additional seizure areas within eloquent cortex (not resected). Intraoperative confirmation of Face motor (F tag), Speech slurring/Mouth motor (Fa tag), Hand motor (A tag). Second post-resection intraoperative photo (**D**) and 3D reconstruction (**E**) show the removed seizure focus.

The patient was seizure free for four months post-operatively. At five months post-operatively, the patient had a cluster of breakthrough seizures. An EEG performed at this time demonstrated frequent generalized spike and wave discharges originating from the left hemisphere and left-hemisphere slowing. Ten months postoperatively at the age of 13, the patient underwent additional workup which led to the hypothesis that the seizures were still originating from the left frontal region, with no suggestion of temporal onset. Repeat surgery was recommended and a 6 × 8 (48-contact) frontotemporal grid was implanted for seizure localization and extraoperative mapping. ECoG showed a diffuse seizure onset starting with rhythmic spike and slow wave activity covering contacts 13–14, 21–22, 29–30, 37–38 and at the depth electrode just posterior to the prior resection (Figure 7). Electrode placement and documentation are as described above and can be seen in Figure 8A–C, with a post-resection image in Figure 8D.

Microscopic examination of the tissue resected at the time of the second surgery revealed disorganized brain consistent with developmental/migrational abnormality. Additional anterior sections of the CST, AF, and medial SLF were removed, while the UF remained preserved. For nine months after his second surgery, the patient was seizure-free. At his most recent follow-up approximately 10 months postoperatively, the patient has had occasional break-through seizures with continuation of his stimulant therapy for ADHD.

**Figure 9:**
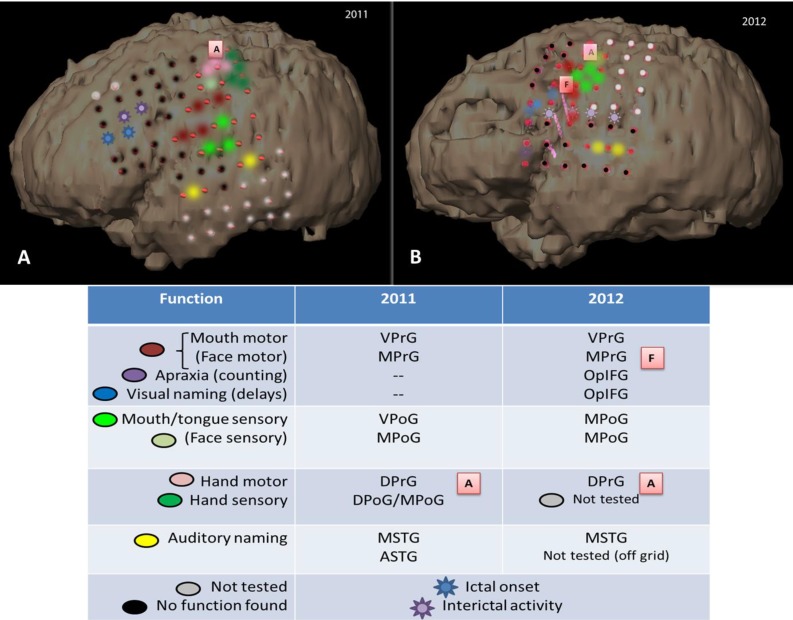
3D reconstructions showing grid placement and function in 2011 (**A**) and 2012 (**B**). In the second map, new areas of speech-motor function are found in the IFG with higher stimulation currents, and tongue-sensory function is reorganized superiorly concomitant with new zones of ictal and interictal activity in the previous tongue-sensory cortical area. Depth electrodes in 2012 map are indicated by pink corkscrews. See legend for color-coded functions and lettered tags.

Cortical areas within the precentral gyrus, primary motor strip, the majority of the sensory strip, and the superior temporal gyrus were mapped across both the 2011 (Figure 9A) and 2012 (Figure 9B) extraoperative sessions. Stimulation currents across comparable areas were generally 3–4 mA higher in 2012 than 2011. Despite these stimulation current differences, several functional areas remained stable across the two maps: (1) no motor, sensory, or language function (using visual naming) within the posterior portions of the superior and middle frontal gyri (PSFG/PMFG); (2) hand motor function in the dorsal portion of the precentral gyrus (DPrG); (3) face/mouth motor function (speech apraxia) in the superior part of the ventral precentral gyrus (VPrG) and middle portion of middle precentral gyrus (MPrG); (4) face sensory function in the anterior/superior portion of middle postcentral gyrus (MPoG); and (5) auditory naming in the middle portion of the superior temporal gyrus (STG). In contrast, various subregions appeared to differ in function within the frontal lobe between 2011 and 2012, including: (1) tongue sensory in the ventral postcentral gyrus (VPoG), appearing to have reorganized superiorly to the MPoG; (2) new visual naming delays in the superior area of the operculum of the inferior frontal gyrus (OpIFG); and (3) new speech arrest within the inferior area of OpIFG.

Patients who have required additional surgery and repeated mapping after a recurrence of seizures appear to undergo little to no change in the localization of language sites that are distant from the margins of resection, which many investigators refer to as the “one centimeter rule” to indicate that a 1 cm margin is needed to avoid inducing a permanent functional deficit [14,15,16,17]. Additionally, a body of work in low-grade glioma (LGG) patients suggests that the preservation of white matter tracts is critical to preserve function despite removal of cortical tissue [5,18]. If both cortical tissue and its underlying white matter are preserved, there would be an expectation of stability in the location of the functional site.

When resections impinge upon language or primary motor areas, however, functional recovery is thought to coincide with not only intrahemispheric, but perilesional reorganization [2,3,4,19,20]. Other clinical variables that could affect reorganization in a pediatric patient include normal age-related maturation processes in the timing and distribution of cortical thinning [21,22,23], myelination of white matter [11], and cerebral metabolism [24]. The frontal lobe outside of motor and sensory areas is known from these studies to have a longer structural and functional developmental trajectory than the temporal lobe, implying that functional areas in the frontal lobe could potentially show more plasticity than areas in the temporal lobe in pediatric patients. However, other mechanisms are also likely to play a role in reorganization, including pathologies inherent in cortical dysplasia and resulting seizure activity.

In the present patient, both stable and reorganized functional areas were identified with extraoperative stimulation prior to the first and second surgical resections. A stable area across the two grid maps was found in the temporal lobe for auditory naming within the middle portion of the STG. Auditory naming errors with cortical stimulation were primarily semantic paraphasias and comprehension errors. The stability of this area can likely be accounted for by its relatively distant location from the first resection, which spared this cortical area, as well as the anterior indirect segment of the superior longitudinal fasciculus (SLF) subcortical tract. This subcortical fiber tract connects the posterior part of the superior temporal gyrus behind Heschl’s gyrus (Wernicke’s area) with the posterior portion of the frontal operculum lateral to the arcuate fasciculus [25]. Although the patient’s Verbal Comprehension Index (VCI) score fell from Average to Borderline between 2005 and 2009 coinciding with an increase in seizure frequency and intractability to anti-seizure medications, the stability of this index following both surgical resections is consistent with preserved cortical and subcortical areas in the posterior STG and anterior segment of the SLF.

Stable areas across the two grid mapping sessions in the frontal lobe were: (1) posterior areas of the SFG and MFG showing no motor, sensory, or language function (using visual naming); (2) DPrG for hand motor function, found both extraoperatively and intraoperatively (Figure 9A,B, tag A); (3) the superior portion of VPrG and middle portion of MPrG showing face/mouth motor function (Figure 9B, tag F); and (4) the anterior/superior portion of the MPoG for face sensory function.

A lack of function as seen by cortical stimulation in the SFG and MFG could be due to a number of factors. Cortical stimulation during visual naming has induced errors in the MFG in a similarly-aged patient [4], though the proximity of this area to an ictal onset (grid map 1), surgical cavity (grid map 2), a low stimulation current (4 mA), or age-related lag of frontal association-area development [26] could each account for either a lack of function or a failure to illicit function through cortical stimulation. In contrast, the SFG is implicated in working memory tasks involving monitoring and manipulation, particularly those involved in spatially oriented processing [27]. Neuropsychological scores in the visual aspect of working memory—which tests visual-spatial imaging and mental manipulation—indicated a dramatic decrease in this function prior to the first surgery, when the patient’s seizures and intractability increased, which is consistent with dysfunction in this area. Although this index score increased after the first resection, possibly reflecting some degree of compensation, scores again decreased following the second resection, which could reflect removal of reorganized cortex perilesionally to the first resection. Since a working memory task was not conducted in this region during cortical stimulation due to time constraints, a lack of function using visual naming tasks was somewhat expected. Overall decreases in the visual Working Memory Index and the Perceptual Reasoning Index lead us to speculate that this area was likely destabilized by cortical dysgenesis [28,29] and that this area did not reorganize a second time following the second surgical resection.

Stable hand motor function in the DPrG and face/mouth motor function in the MPrG/VPrG may be accounted for by their relative distance from the first resection (>2.5 cm and 2 cm, respectively) as well as the preservation of corticospinal tracts (CST) that project into these areas of primary motor cortex. As the head of the caudate nucleus and the frontal horn of the lateral ventricle were spared, the internal capsule fibers passing through the corona radiata into these areas of motor cortex [30] were also likely left intact. Cortical areas for face sensory function also remained stable between the two functional maps, which is likely accounted for by the sparing of subcortical fibers running from this area of the cerebrum to the ventral posterior medial thalamic nucleus and beyond to the trigeminal lemniscus.

The main areas that appear to differ in function across the two grid maps include: (1) tongue sensory in the ventral postcentral gyrus (VPoG), which reorganized superiorly to the middle post-central gyrus (MPoG); (2) visual naming hesitations in the superior area of the operculum of the inferior frontal gyrus (OpIFG); and (3) speech arrest within the inferior area of OpIFG. Motor and sensory areas are some of the earliest to mature developmentally and in terms of grey matter volume, maturing earlier than temporal lobe association areas [26]. Given the stability of auditory naming sites in the posterior STG—an area that matures later than frontal motor and sensory areas—age-related changes are not likely to account for the shift in sensory areas. The tongue sensory area in each map is >2 cm away from the first resection, making surgically-induced changes in cortical or subcortical areas also unlikely to account for the change in location. However, an interval change in the location of the epileptogenic zone was found, with epileptigenic activity shifting posteriorly and inferiorly from anterior/middle MFG to the operculum of the IFG, extending posteriorly to VPrG and VPoG. With this shift in the epileptic zone came a directly superior shift in tongue/mouth sensory function from the VPrG and VPoG to the lower portion of the MPoG. Reorganization of primary somatosensory area in epilepsy associated with cortical dysplasia in a pediatric patient has previously been reported, showing reorganization of the left thumb area into a restricted area of normal frontal lobe adjacent to the dysplastic brain [31,32], as has reorganization of the motor hand area in an older teen (19 years) with cortical dysplasia [3]. The source of spikes observed on EEG remains controversial, with evidence pointing both to surrounding cortex [33] as well as over the lesion itself [34]. In the present patient it appears that interictal spikes originated over normal cortex adjacent to the lesion, suggesting an irritative zone that may have promoted reorganization of the tongue sensory area to its new location. Newly found areas in the operculum of the inferior frontal gyrus include both visual naming delays and speech arrest, the former located immediately superior to the latter within this subregion, which is classically identified as Broca’s area. Speech arrest and hesitations in this area are associated more with a speech-motor function than a frontal language function. This is consistent with visual naming deficits being relatively rare in frontal lobe epilepsy pediatric patients [35] and the location of visual naming errors during cortical stimulation found more superiorly in the MFG [4], likely as a result of terminations of the arcuate fasciculus, which is implicated in various aspects of language [25,36]. Together, these visual naming delays point to a speech-motor function with an earlier developmental profile and away from age-related functional changes. The presence of afterdischarge activity in this area during the first grid mapping limited the stimulation current given (4 mA), so it is possible that the current was too low to elicit disruption of this function and produced a false-negative compared with a relatively higher stimulation current (7 mA) used in the second mapping.

Neuropsychological profiles of this patient are likely accounted for by a combination of surgical resections as described above and known cognitive consequences of frontal lobe epilepsy. Prior to the first surgical resection, declines in VCI, PSI, WMI, and FSIQ were seen, which is consistent with profiles seen in frontal lobe pediatric epilepsy studies [35,37,38,39]. The wide range of cognitive impairment prior to surgical intervention may be the result of rapid propagation of epileptic discharges both inter- and intra-hemispherically [38] or from subcortical abnormalities that prevent efficient communication between cortical areas [40]. Following surgical interventions, declines continued in auditory attention, visual memory, and executive functions. Executive functions such as planning, attention, organization, and motor coordination, as well as visuo-spatial attention and visuomotor integration, which are all mediated by the frontal lobe, have been known to decline following surgical intervention in lesional frontal lobe pediatric patients [35], potentially resulting from a combination of both cortical and subcortical resections.

## 3. Experimental Section

### 3.1. Fiber Tracking

DTI MR images were obtained following the first resection in 2012 with a 3.0-T system (SignaHDxt, GE Healthcare, Waukesha, WI, USA) using an echo-planar sequence (12,000/91.5/1 [TR/TE/NEX]), 21-cm field of view, matrix 92 × 92, 2.5 mm section thickness, b = 0/1000 s/mm^2^, with diffusion encoding in 25 directions.

Tractography was performed on a dedicated workstation (Advantage Windows workstation, GE Healthcare, Waukesha, WI, USA) using commercially available fiber tracking software (FuncTool version 9.4.05a, GE Healthcare, Waukesha, WI, USA). The white matter tracts were defined by manually placing a seed region of interest in mirrored locations in the right and left hemisphere based on known tract locations [41]. Tract propagation was then carried out in both antegrade and retrograde directions, using a minimum FA value of 0.18.

### 3.2. Mapping of Motor, Sensory, Speech-Motor, and Language Function

Parental consent and child assent were obtained in accordance with the guidelines of the Internal Review Board of Duke University Health System (Pro00004155, 11/26/2002 and Pro00020555, 11/02/2009). Testing prior to the 2011 resection was performed in two sessions over two days. The area of exposure included frontal, temporal, and parietal areas. Eight electrodes in the frontal grid (21, 22, 23, 24, 29, 30, 31, 32) and eight electrodes in the temporal grid (16, 17, 18, 20, 21, 22, 23, 24) were tested in pairs on the first day using visual and auditory naming (see Supplementary Information for stimuli). Forty-six electrodes in the frontal grid (1–46) were tested in pairs on the second day for motor, sensory, and speech-motor functions. Testing prior to the 2012 resection was performed in a single session. The area of exposure was similar, including frontal, temporal, and parietal areas. Thirty-six electrodes were tested in pairs for motor, sensory, and speech-motor functions and using visual and auditory naming. Baseline performance was measured by pre-testing all tasks several days prior to grid placement. Only items that the patient successfully completed at least twice each at baseline during the testing were administered during cortical mapping. The reliability of errors for each site in each task was calculated relative to the unstimulated baseline error rate using the Fisher Exact Test and a significance level of 0.05.

To ensure that receptive language was not compromised during testing, stimulation was administered following presentation of the picture (visual naming) or auditory definition (auditory naming). Before each response, the patient spoke the carrier phrase “This is a …” to ensure stimulation was not causing a general speech arrest. The presentation of the picture was accompanied by an aural cue with the presentation software, alerting the neurosurgeon that the stimulus was on the screen. Stimulation was initiated immediately following this aural cue with a delay of approximately 1000 ms, with the stimulus on the screen for 5000 ms. Stimulation for auditory naming was initiated immediately after the beginning of the definition without an additional aural cue. The short delay after the appearance of the stimulus allowed stimulation to proceed immediately after the stimulus was on the screen or began to be heard but before the patient began his oral response of the carrier phrase and stimulus item.

The following error types [42] were noted: (1) semantic paraphasias (for example, substituting a semantically related item such as “chair” for “table”); (2) phonological paraphasias (for example, clear substitution of one phoneme for another such as /f/ for /v/ or /tr/ for /dr/); (3) semantic/phonological blends (for example, substituting “train” for “plane”); (4) off-target responses, that is those responses not semantically or phonologically related to the correct response (for example, substituting “tree” for “fork”); (5) no-target responses, that is, the patient correctly says the carrier phrase but is unable to give a response to the stimulus; (6) perseveration, in which the patient responds to a previous stimulus within five trials; (7) apraxic errors such as a slur or stutter; (8) phonological reduction, in which a syllable is dropped from a word (for example, responding with “can” instead of “candle”); (9) neologism, where the response is a pronounceable non-word, such as “zobluch” for “kite”, and (10) comprehension error, where the patient was unable to comprehend the definition presented aurally during stimulation.

## 4. Conclusions

In summary, widespread and significant cognitive, memory, and IQ declines were seen during a period of increased seizure frequency and intractability to antiepileptic medications prior to the first surgical resection. The two cortical grid maps prior to each resection showed stability in posterior temporal regions that involved auditory naming, consistent with the preservation of cortical areas and SLF subcortical tracts in this region, and as further reflected in verbal domains of neuropsychological testing scores. Grid maps also showed stability in motor areas of face/mouth and hand, also consistent with CST subcortical preservation that projected to these regions. Conversely, areas of reorganization were seen in cortical tongue sensory areas concomitant with a new zone of ictal and interictal activity in the previous tongue sensory area. Increased current stimulation during the second grid mapping most likely accounts for new areas of speech-motor function found in the IFG. Additional cortical and subcortical tract removal (anterior AF and medial SLF) anterior to the motor strip in the second resection appears to have led to additional declines in visual working memory and attention measures in the presence of lowered seizure frequency.

## Supplementary Files

Supplementary File 1 Supplementary Information (PDF, 925 KB)
