# Supplementary material for: Reorganization and Stability for Motor and Language Areas Using Cortical Stimulation: Case Example and Review of the Literature
Source: Brain Sci. 2013 Nov 26;3(4):1597–614. doi: 10.3390/brainsci3041597 (PMC4061887; doi:10.3390/brainsci3041597)
Supplement: Supplementary File 1 — Supplementary Information (PDF, 925 KB) [file brainsci-03-01597-s001.pdf]

Supplementary Information

Part 1

Wechsler Intelligence Scale for Children—Fourth Edition

The Wechsler Intelligence Scale for Children—Fourth Edition (WISC-IV) is an individually administered clinical instrument for assessing the cognitive skills of children age 6 years 0 months through 16 years 11 months. It is comprised of 15 subtests, each measuring various facets of intelligence. Ten subtests are routinely administered to calculate the four index scores and the Full Scale IQ.

The theoretical foundation of the WISC-IV is derived from Wechsler's original Verbal-Nonverbal theory of intelligence (1939). The WISC-IV reflects an increased attention to the importance of working memory and processing speed in the importance of cognitive functioning. A complete administration of the WISC-IV yields standard scores in the following composite areas:

Verbal Comprehension Index
Perceptual Reasoning Index
Working Memory Index
Processing Speed Index
Full Scale IQ

Following are descriptions of each of the ten core WISC-IV subtests:

WISC-IV Verbal Comprehension Subtests

Similarities measures verbal reasoning and concept formation. It also involves auditory comprehension, memory, distinction between nonessential and essential features, and verbal expression. This test requires the child to identify the similarity between two ostensibly dissimilar items (words presented orally to the child).

Vocabulary measures the child's word knowledge and verbal concept formation. For the picture items, the child is required to name the pictures in the stimulus book. For the verbal items, the child is required to give definitions for words the examiner reads aloud.

Comprehension measures the child's verbal reasoning and conceptualization, verbal comprehension, and verbal expression. This test requires the child to answer questions based on his or her understanding of general principles and social situations.

WISC IV Perceptual Reasoning Subtests

Block Design measures the child's ability to analyze and synthesize abstract visual stimuli. This test requires the child to view a constructed model or a picture in the stimulus book, and use red-and-white blocks to re-create the design within a specified time limit.

Picture Concepts measures abstract, categorical reasoning ability. The child is presented with two or three rows of pictures and chooses one picture from each row to form a group with a common characteristic.

Matrix Reasoning measures fluid intelligence and provides a reliable estimate of general nonverbal intelligence. This test requires the child to view an incomplete matrix and select the missing portion from 5 response options.

WISC IV Working Memory Subtests

Digit Span measures auditory short-term memory, sequencing skills, attention, and concentration. The Digit Span Forward task requires the child to repeat numbers in the same order as read aloud by the examiner. Digit Span Backward requires the child to repeat the numbers in the reverse order of that presented by the examiner.

Letter-Number Sequencing measures sequencing, mental manipulation, attention, short-term auditory memory, visual-spatial imaging, and processing speed. It requires the child to read a sequence of letters and numbers and recall the numbers in ascending order and the letters in alphabetical order.

WISC IV Processing Speed Subtests

Coding measures the child's short-term memory, learning ability, visual perception, visual-motor coordination, visual scanning ability, cognitive flexibility, attention, and motivation. It requires the child to copy symbols that are paired with simple geometric shapes or numbers.

Symbol Search measures processing speed, short-term visual memory, visual-motor coordination, cognitive flexibility, visual discrimination, and concentration. This test requires the child to scan a search group and indicate whether the target symbol(s) matches any of the symbols in the search group within a specified time limit.

WRAML2 Subtests

The WRAML2 is a reliable, norm-referenced test that has been nationally standardized to assess a wide range of clinical issues related to learning and memory functions.

The WRAML2 is composed of six core subtests that yield three indexes: (a) the Verbal Memory Index, (b) the Visual Memory Index and (c) the Attention/Concentration Index. Together, these indexes form the General Memory Index, which is a composite of all assessed memory functions. In addition, there are several optional subtests and diagnostic procedures.

It is recommended that the WRAML2 be administered in its entirety to improve the reliability and the validity of the test results. However, time constraints sometimes may dictate that only portions of the WRAML2 can be administered. Accordingly, less confidence in results should be assumed when only a portion of the WRAML2 is administered. As indicated in the WRAML2 Administration and Technical Manual, reliability is highest for the General Memory Index, followed by the individual index scores and, lastly, by the individual subtest scores. When there are significant differences between index scores or when there is significant variability between subtests within an index, caution is required in interpreting the respective index, including the General Memory Index. It is important to note that the standard scores presented in this report are based on a mean of 100 and a standard deviation of 15, and scaled scores presented in this report are based on a mean of 10 and a standard deviation of 3. In addition, the following qualitative classification ranges have been created for both

standard scores [Very Superior (>129), Superior (120–129), High Average (110–119), Average (90–109), Low Average (80–89), Borderline (70–79), and Impaired (<70)] and scaled scores [High Average (>12), Average (8–12), Borderline/Low Average (4–7), and Impaired (<4)].

Verbal Memory Index is an estimate of how well the client can learn and recall both meaningful verbal information and relatively rote verbal information. It is derived from the sum of the Story Memory subtest and the Verbal Learning subtest. When consistent performance exists between the two subtests comprising this index, the index presents a reasonable estimate of verbal memory abilities. More specifically, Verbal Memory Index performance is correlated with abilities for everyday tasks (e.g., remembering stories, conversations, or information from lectures; following directions; recalling items from a "things to do" list). Related academic tasks can include the ability to recall the content of information that was read earlier, the ability to learn lists of scientific terms, or the ability to remember vocabulary words.

Visual Memory Index is an estimate of how well the client can learn and recall both meaningful (*i.e.*, pictorial) and minimally related, rote (*i.e.*, design) visual information. It is derived from the sum of the Picture Memory subtest and the Design Memory subtest. When consistent performance exists between the two subtests comprising this index, the index presents a reasonable estimate of visual memory ability. More specifically, visual memory abilities may be related to day-to-day tasks (e.g., remembering the layout of the town visited a while ago, identifying different car models, remembering the location of states on a map). Related academic tasks can include the recall of information from the chalkboard, some aspects of math problems (e.g., graphs, spatial problems), and processing/recalling less verbal or nonverbal aspects of science/technology like a circuit diagram).

Woodcock-Johnson Tests of Cognitive Abilities-III (WJ-III COG) is an individually administered battery of tests of cognitive abilities designed for ages 2 to 90 years. Three individual subtests were administered, two of which create a composite score for Auditory Processing.

Auditory Processing involves the ability to analyze, synthesize, and discriminate auditory stimuli. The *Auditory Attention* subtest is a measure of speech-sound discrimination, including the ability to overcome the effects of auditory distortion (e.g., unrelated sounds) or masking in understanding oral language. On this subtest the child listens to a word, while seeing four pictures, and is asked to point to the correct picture for the word, with increasing background sound.

Sound Blending is an auditory processing test of phonetic coding that measures skill in synthesizing language sounds (phonemes). During this subtest, children listen to an audio presentation of separate phonemes and are then asked to blend sounds into a word.

Rapid Picture Naming is a test of cognitive fluency and facility with naming objects. For this subtest the child is asked to name as many pictures as he/she can within a limited time frame.

Part 2

Cortical Stimulation Stimuli—Auditory and Visual Naming

Table S1. Target names and auditory naming stimuli presented to patient.

Target Name	Auditory Naming Stimulus
Balloon	You blow it up at birthday parties
Banana	A monkey's favorite yellow fruit
Bed	Furniture for sleeping on
Belt	It's buckled around the waist
Broom	Used to sweep floors
Candle	It's lit on birthday cakes
Cat	A pet that meows
Crayon	You color pictures with it
Crown	Kings wear them on their head
Dog	A pet that barks
Duck	A bird that quacks
Football	Used to throw a touchdown
Hammer	A tool to hit nails
Horse	Cowboys ride them
Moon	Astronauts walked on it
Mouth	A body part for talking
Nose	A body part for smelling
Popsicle	Frozen treat on a stick
Potato	A vegetable used to make French fries
Pumpkin	It's carved for Halloween
Spider	An eight-legged insect that spins webs
Spoon	You eat soup with it
Telephone	It rings when someone calls
Toothbrush	It's used to clean your teeth

Table S2. Target names and visual naming stimuli presented to patient.

Target Name	Visual Naming Stimulus	Target Name	Visual Naming Stimulus
Airplane	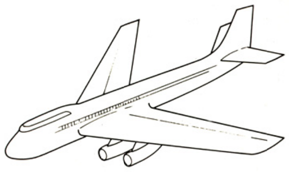	Fork	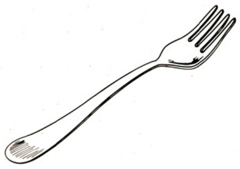
Apple	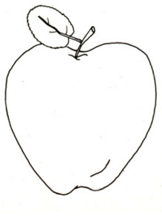	Frog	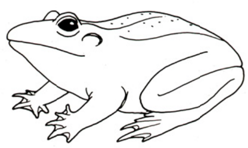

Table S2. *Cont.*

Target Name	Visual Naming Stimulus	Target Name	Visual Naming Stimulus
Balloon	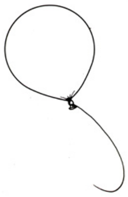	Hand	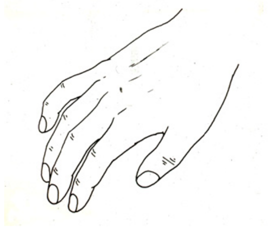
Banana	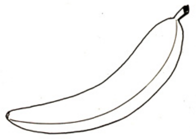	Heart	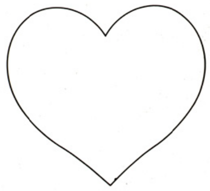
Bat	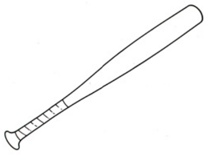	Kite	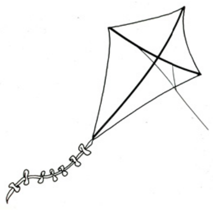
Bed	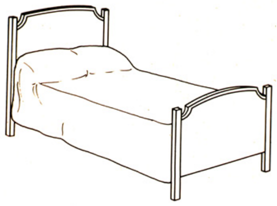	Lightbulb	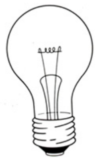
Bird	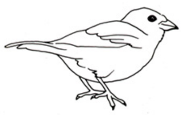	Mountain	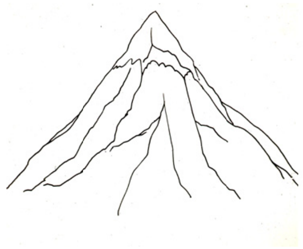
Book	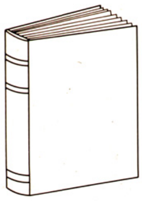	Nose	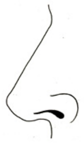

Table S2. *Cont.*

Target Name	Visual Naming Stimulus	Target Name	Visual Naming Stimulus
Broom	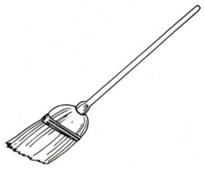	Pencil	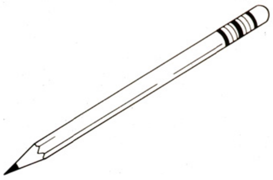
Candle	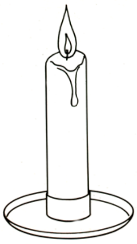	Pig	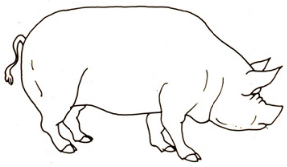
Cat	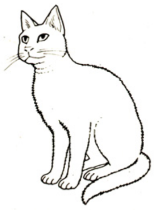	Rabbit	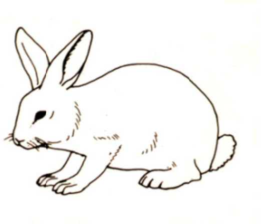
Chair	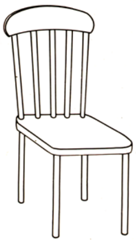	Shoe	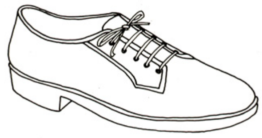
Comb	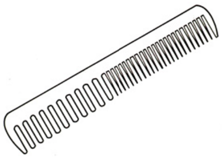	Spoon	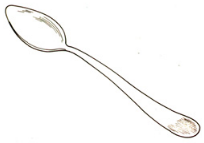
Dog	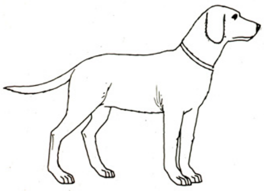	Star	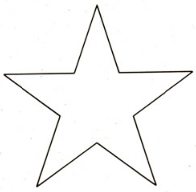

Table S2. *Cont.*

Target Name	Visual Naming Stimulus	Target Name	Visual Naming Stimulus
Duck	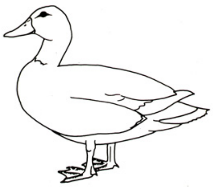	Sun	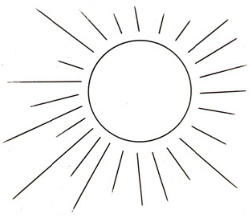
Eye	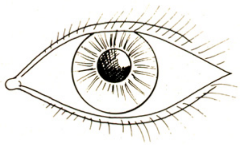	Table	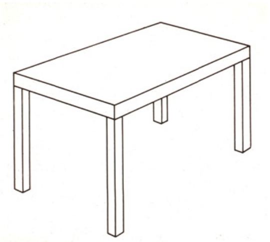
Finger	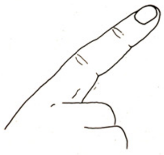	Toothbrush	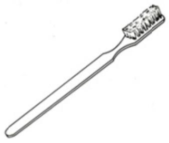
Fish	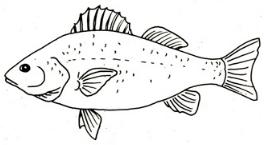	Turtle	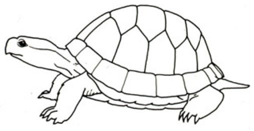
Flower	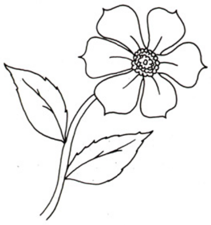	Umbrella	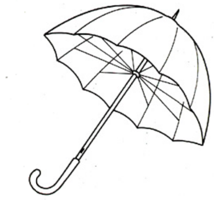

Table S2. *Cont.*

Target Name	Visual Naming Stimulus	Target Name	Visual Naming Stimulus
Foot	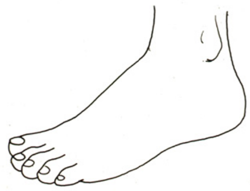		

© 2013 by the authors; licensee MDPI, Basel, Switzerland. This article is an open access article distributed under the terms and conditions of the Creative Commons Attribution license (http://creativecommons.org/licenses/by/3.0/).
